# Spatiotemporal dynamics and policy impact on farmer suicides in Maharashtra, India

**DOI:** 10.1038/s41598-025-03335-7

**Published:** 2025-06-01

**Authors:** Anjali, B. Rushi Kumar

**Affiliations:** Department of Mathematics, School of Advanced Sciences, Vellore Institute of Technology, Vellore, Tamil Nadu 632014 India

**Keywords:** Spatiotemporal analysis, Logistic regression, Relative risk, Farmer’s suicide, Public health, Suicide prevention, Risk factors, Mathematics and computing

## Abstract

Maharashtra has long faced a severe agricultural crisis, with a high number of farmer suicides, making it crucial to evaluate how government agricultural policies affect farmer suicides. This study investigated the influence of the Pradhan Mantri Fasal Bima Yojana (PMFBY), a crop insurance scheme, on patterns of farmer suicides across the state. Data for this study is acquired from the “Crime in Maharashtra” and the “Pradhan Mantri Fasal Bima Yojana (PMFBY) scheme” report spanning 2017–2021. This study focuses on analyzing the spatiotemporal trends to identify suicide-prone regions and assess whether the PMFBY has mitigated this distress. Further, this study employs statistical techniques to highlight key factors influencing suicides in these clustered regions. The findings suggest a notable reduction in suicide occurrences following the implementation of PMFBY, especially in high-risk districts. The study highlights that the districts of Aurangabad, Nagpur, and Amravati divisions emerged as significant clusters during the period from 2017 to 2019, and predicted rainfall, insurance units, gender-specific distribution, and agricultural land as positively influencing in mitigating the occurrence of farmer suicides within these clustered regions. Overall, this research underscores a notable reduction in the occurrence of both concentrated and dispersed regions before and after the introduction of the PMFBY. This study offers insights to policymakers for designing more targeted interventions aimed at reducing agrarian distress, improving farmer well-being, and offering valuable insights to improve suicide prevention initiatives in Maharashtra’s districts and more broadly across India.

## Introduction

### Background

The idyllic images of rural landscapes and bountiful harvests often overshadow a disheartening reality: a distressing increase in farmer suicides^1,2^. Even in the absence of mental health issues, farmers express a sense of hopelessness more frequently than urban residents do. They resort to suicide due to an accumulation of persistent and overwhelming challenges^3,4^. This dire situation underscores a profound crisis within the agricultural sector, where farmers, often burdened by debt, inadequate access to resources, market instability, crop failures, the adverse effects of climate change, and economic hardships, resort to taking their own lives^5,6^. Farmer suicides are a tragic and intricate issue observed in various parts of the world^7,8^. The prevalence of these suicides varies significantly by region and is influenced by a complicated interaction of economic, social, and environmental factors^9,10^.

Agriculture has been the cornerstone of India’s economy, providing the means of subsistence for countless families across the country^11,12^. Unfortunately, in recent years, the distressing truth is that farmer suicides have become alarmingly common, with an estimated 10,000 farmers and those reliant on agriculture taking their own lives each year^13,14^. The most recent data released by the National Crime Records Bureau (NCRB) reveals that in 2020, there were 10,677 instances of suicide in the agricultural sector, constituting 7% of all suicides in India^15^. This issue is particularly prominent in states heavily dependent on agriculture. Maharashtra has consistently recorded a high number of farmer suicides, followed by Karnataka, Andhra Pradesh, and Telangana^16,17^.

The “Crime in Maharashtra” report for 2019 painted a bleak picture, with 2680 farmers sadly resorting to suicide^18^. Agricultural suicides in Maharashtra result from a combination of factors, which include accumulated debt from loans taken for farming expenses, financial pressures, unpredictable weather patterns, such as droughts, untimely rains, and pest infestations, leading to crop failures and substantial financial setbacks for farmers, limited access to formal credit and financial services, compelling farmers to resort to informal sources of credit, often with high-interest rates, and income uncertainty due to market fluctuations in crop prices, which can be unpredictable and affect a farmer’s earnings^19,20^. The agricultural crisis in India, specifically in Maharashtra has emerged as a significant cause for concern, prompting the government to introduce various initiatives to address this issue and discuss agricultural reforms and rural development^21,22^.

Numerous government initiatives have been introduced over the years to ameliorate the conditions of Indian farmers. One of the latest initiatives is the PMFBY, which was introduced through the joint effort between the Department of Agriculture, Cooperation and Farmers Welfare (DAC & FW) and various other organizations under the Ministry of Agriculture and Farmers Welfare (MoA & FW)^23^. The primary goal of PMFBY is to provide economic security and insurance coverage to farmers in case they suffer crop damage due to natural calamities, pests, or diseases^24^.

The PMFBY scheme was launched to offer financial protection to farmers by compensating for losses caused by various economic, social, and agricultural challenges that directly or indirectly may lead to distress and suicidal tendencies. From a psychological perspective, the assurance of crop insurance provides farmers with a sense of security, easing anxiety about agricultural risks. Additionally, the scheme stabilizes farm incomes, enabling farmers to plan for future agricultural activities and meet basic family needs, thereby reducing financial uncertainty. With the confidence of insurance coverage, farmers are encouraged to invest in improved agricultural practices, potentially boosting productivity and profitability.

The unexplored statistical relationship between the Pradhan Mantri Fasal Bima Yojana (PMFBY) and the incidence of suicides in various regions of Maharashtra highlights a significant gap in understanding the scheme’s broader socio-economic impact. Recognizing this gap, we were motivated to delve deeper into the interplay between PMFBY’s financial protection mechanisms and the structural reforms it incorporates. Our exploration seeks to understand how these multifaceted interventions under PMFBY contribute to addressing the pressing issue of agrarian suicides. By analyzing this relationship, we aim to shed light on the scheme’s potential to alleviate the economic vulnerabilities of farmers and mitigate the stressors that lead to such tragic outcomes.

While several studies have examined farmer suicides and the impact of policy interventions, few have employed a spatiotemporal analytical lens to explore how these patterns evolve across both geography and time. Spatiotemporal analysis offers a powerful methodological advantage by detecting statistically significant suicide clusters and tracking their movement across space and time. This dynamic view allows researchers and policymakers to identify not only where and when suicide risks are elevated but also how these risks respond to the implementation of schemes like PMFBY. By integrating spatial and temporal dimensions, our methodology bridges a crucial gap in policy evaluation-moving beyond static or aggregate assessments to uncover localized, time-sensitive trends. This enables a more nuanced understanding of whether targeted interventions are reaching the most vulnerable regions and how their impact unfolds over time, thereby offering actionable insights for region-specific policy refinement.

### Related work

This literature review, grouped into thematic sections based on the type of studies and focus areas, explores the identification and analysis of suicide clusters through spatial and spatio-temporal methodologies across various domains. It emphasizes global and Indian perspectives, linking suicide patterns to social, environmental, economic, and policy-related factors. The review also highlights the role of government initiatives in mitigating suicide risks, particularly among vulnerable farming communities.

#### Mental health, environment, and social determinants of suicide

Farming has been ranked as one of the most difficult industries by studies all across the world. Prior studies have found a link between financial and farming issues with suicide. Farmers in the United Kingdom were more concerned about family problems^25^, while debt and financial worries were cited as the leading causes of suicide among Indian farmers^26,27^. A retrospective cohort study that used linked Swedish national registers, geospatial analysis, and multilevel logistic regression models to examine the potential impact of neighborhood crime on the odds of major depression, showed that people living in areas with high levels of neighborhood crime are more likely to experience major depression^28^. Zhang et al.^29^ examined and suggested that urban flow, water-bodies, greenery, and places to sit are linked to stress response changes. In a novel approach, Sung et al.; Roy et al.^30,31^ designed a model-based suicide risk index using Bayesian spatial factor analysis to assess and identify area-level suicide risk. This study found that greater deprivation and social fragmentation were positively associated with agrarian suicide risk.

#### Understanding suicide clusters and the need for early detection

A suicide cluster is generally described as an uncommonly high occurrence of suicidal actions happening in a relatively close time and location, surpassing what would normally occur by chance^32^. Nevertheless, significant community anxiety exists regarding these clusters because they can perpetuate themselves. In recent times, the identification and tracking of suicide clusters have gained more attention^33,34^. This effort enables early interventions in these cluster areas to deter further harm and loss of life.

#### Spatiotemporal methods in public health and social science

Globally, extensive research has been conducted across diverse geographical regions and time periods, employing various methodologies to identify spatial and spatiotemporal clusters across multiple domains and datasets. These investigations span a wide range of fields, including criminology, agriculture, environmental studies, urban planning, epidemiology, and public health^35^. The methodologies employed in these studies range from spatial statistical tools like Moran’s I, Geary’s C, and Getis-Ord Gi* to advanced machine learning techniques and Geographic Information Systems (GIS)^36^. By integrating spatial and temporal dimensions, these analyses provide deeper insights into patterns and trends, enabling policymakers, researchers, and stakeholders to design evidence-based solutions tailored to specific regional and temporal contexts^37^.

#### Suicide in India and spatiotemporal analysis

In India, studies have identified criminal hotspots with high levels of criminal activity, using spatial scan-statistics^38,39^. For suicide clusters, Too et al.; P. K. Saravag and B. R. Kumar; Anjali and B. R. Kumar^40–42^ used the spatial and spatiotemporal approach with Poisson discrete scan statistics to discover major hotspots of suicides. Other than these, some studies deeply study suicide incidences in India and other states of India^43,44^. For Maharashtra, several researchers have examined the causes of farmer distress and proposed coping strategies, with a particular focus on climate change-induced suicides in the Vidarbha and Marathwada regions, as well as the overall trends and underlying factors contributing to farmer suicides across the state^45^. Similarly, Tikadar and Kamble^46^ identified indebtedness, low productivity, crop failure, low income, more expenditure than income, and inability to fulfill family requirements as the main causes of farmer suicide in the Marathwada region of Maharashtra. Further, Kumar et al.; Dhawle and Narkar^47,48^ conducted a detailed study in India and other parts of India, and found that cropping, rainfall, and irrigation patterns were significantly correlated with farmer suicide.

#### Government schemes evaluation and suicide prevention efforts

Some studies, Kumara and Kumari; Mandavi et al.^49,50^, have assessed the effectiveness of the PMFBY scheme across India and for a particular region^51–53^. These studies reveal substantial variations in the proportion of beneficiaries among regions, with Western states having a 45.4% ratio, while North-eastern states only have 3.13%^54,55^. Moreover, Gupta; Sulaiman and Murigi; Swain et al.; Shrimali et al.^56–59^ analyzed the impact of government initiatives, particularly PMFBY, on reducing farmer suicides across different regions and states in India.

Despite numerous studies exploring the socio-economic causes of farmer suicides, there remains a significant gap in evaluating the geographical and temporal dimensions of these incidents, particularly in relation to policy interventions like the PMFBY. Traditional approaches often overlook the localized and time-bound nature of suicide patterns, which limits their ability to inform region-specific strategies. Spatiotemporal analysis offers a robust methodological advantage by enabling the identification of suicide clusters across both space and time. By applying discrete Poisson scan statistics through SaTScan software, this study goes beyond aggregated suicide counts to detect high-risk districts and periods where suicide incidences are unusually concentrated. This approach provides a more granular evaluation of PMFBY’s effectiveness, allowing for the assessment of whether policy implementation corresponds with a measurable shift in suicide patterns. In doing so, this methodology bridges the gap between macro-level policy formulation and micro-level impact assessment, offering critical insights for tailoring interventions based on district-specific vulnerabilities and temporal trends.

While previous research has explored the spatial distribution of various aspects in different disciplines related to Maharashtra^60,61^, there exists a notable absence of literature specifically dedicated to examining both the spatiotemporal aspects of farmer suicide and conducting a statistical assessment of the Pradhan Mantri Fasal Bima Yojana (PMFBY) within the context of government initiatives aimed at supporting farmers. Recognizing this research gap, this study is designed to tackle the pressing issue of farmer suicide in the state of Maharashtra, India, and seeks to answer the following research questions:

- What are the spatiotemporal patterns of farmer suicides before and after PMFBY’s implementation?
- Do core variables linked to PMFBY show correlations with suicide incidences in identified high/low risk areas?
- To what extent do socio-demographic and environmental factors contribute to the clustering of farmer suicides?

## Study material and methods

The study’s research framework involved a retrospective examination of all agrarian suicides that took place in the Maharashtra districts, focusing on population-based data. It employed geographic coordinates (longitude and latitude) to analyze the location and time of each suicide event. Using a Poisson Discrete model for scan statistics, the investigation aimed to pinpoint statistically significant suicide clusters in specific districts and time frames.

The paper is organized into three sections. The first section offers a comprehensive overview of suicide occurrences between 2017 and 2021 in Maharashtra’s districts, as well as the government initiative PMFBY during 2018–2021. In the second section, we delve into the detection of spatiotemporal clusters of suicides during two phases of the PMFBY scheme: the initial implementation phase (Phase 1, spanning from 2017 to 2019) and the subsequent operational phase (Phase 2, covering 2019 to 2021). Lastly, the third section employs an in-depth logistic regression approach to compare suicide incidents within clusters and those occurring outside of clusters in Maharashtra’s districts, to identify the key contributing factors corresponding to the PMFBY scheme.

### Geographical region under study

This research focuses primarily on Maharashtra, as it has consistently recorded the highest number of farmer suicides in India over recent years, followed by Karnataka, Andhra Pradesh, and Telangana.

Maharashtra, covering a total area of 307,713 square kilometers (118,809 square miles), is the third-largest state in India by land area, accounting for 9.36 percent of the country’s total geographical area. It lies between latitudes $15^{\circ }$ 35′ N and $22^{\circ }$ 02′ N and longitudes $72^{\circ }$ 36′ E and $80^{\circ }$ 54′ E, occupying the western and central regions of the country. The state has an 840-kilometer-long coastline along the Arabian Sea, with its dominant geographical feature being plateau terrain, separated from the Konkan coast by the Western Ghats mountain range, which runs parallel to the coast from north to south.

**Fig. 1:**
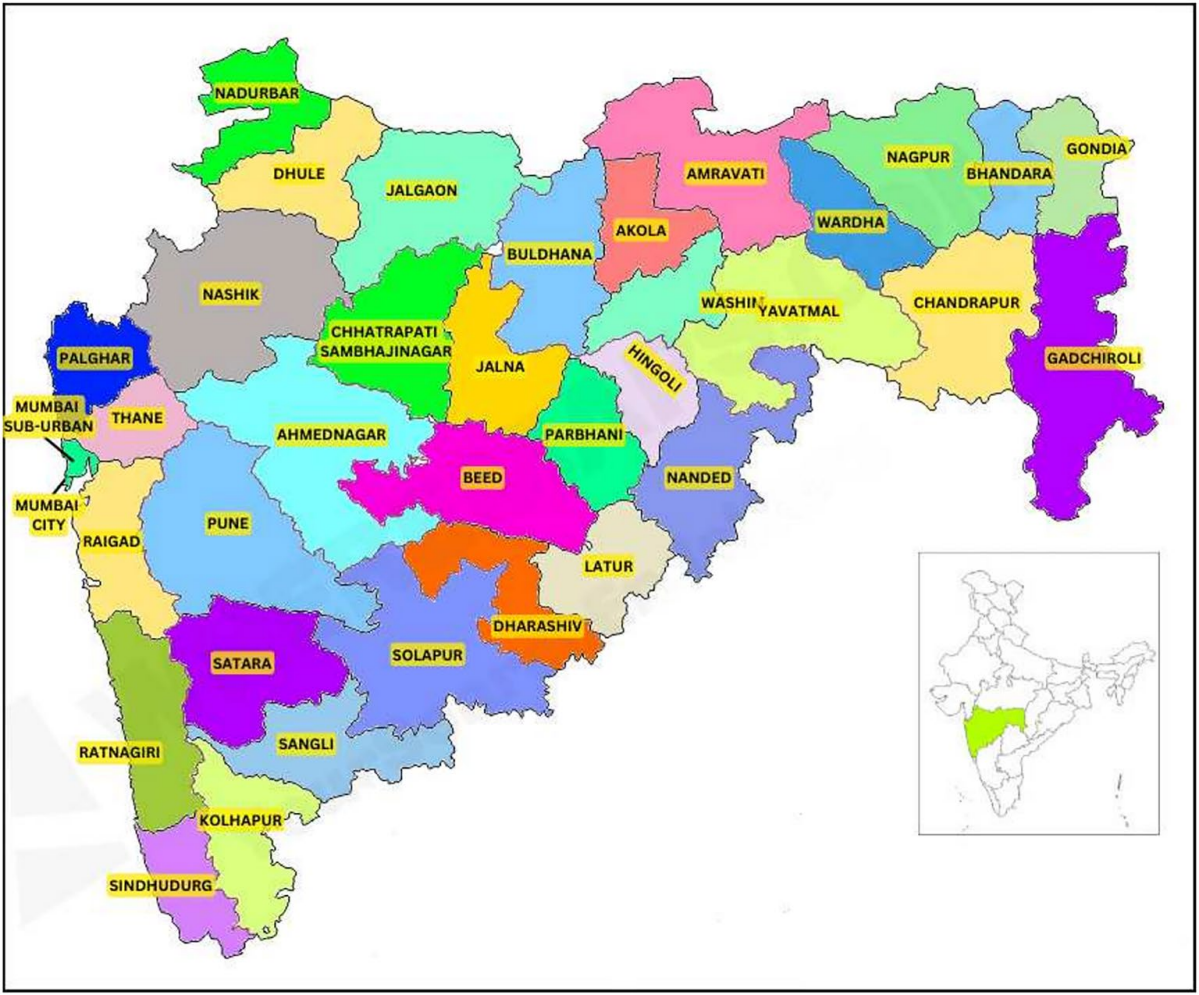
Study region (District of Maharashtra State, India) (Acquired from Google images, https://wheremaps.com/india/maharastra/maharashtra-district-map.html).

It is segmented into six divisions and thirty-six districts. This research primarily concentrates on the districts within the Maharashtra region. Figure 1 offers a geographical map displaying all the districts to enhance comprehension of the study area for this research work.

### Data source and design

**Fig. 2:**
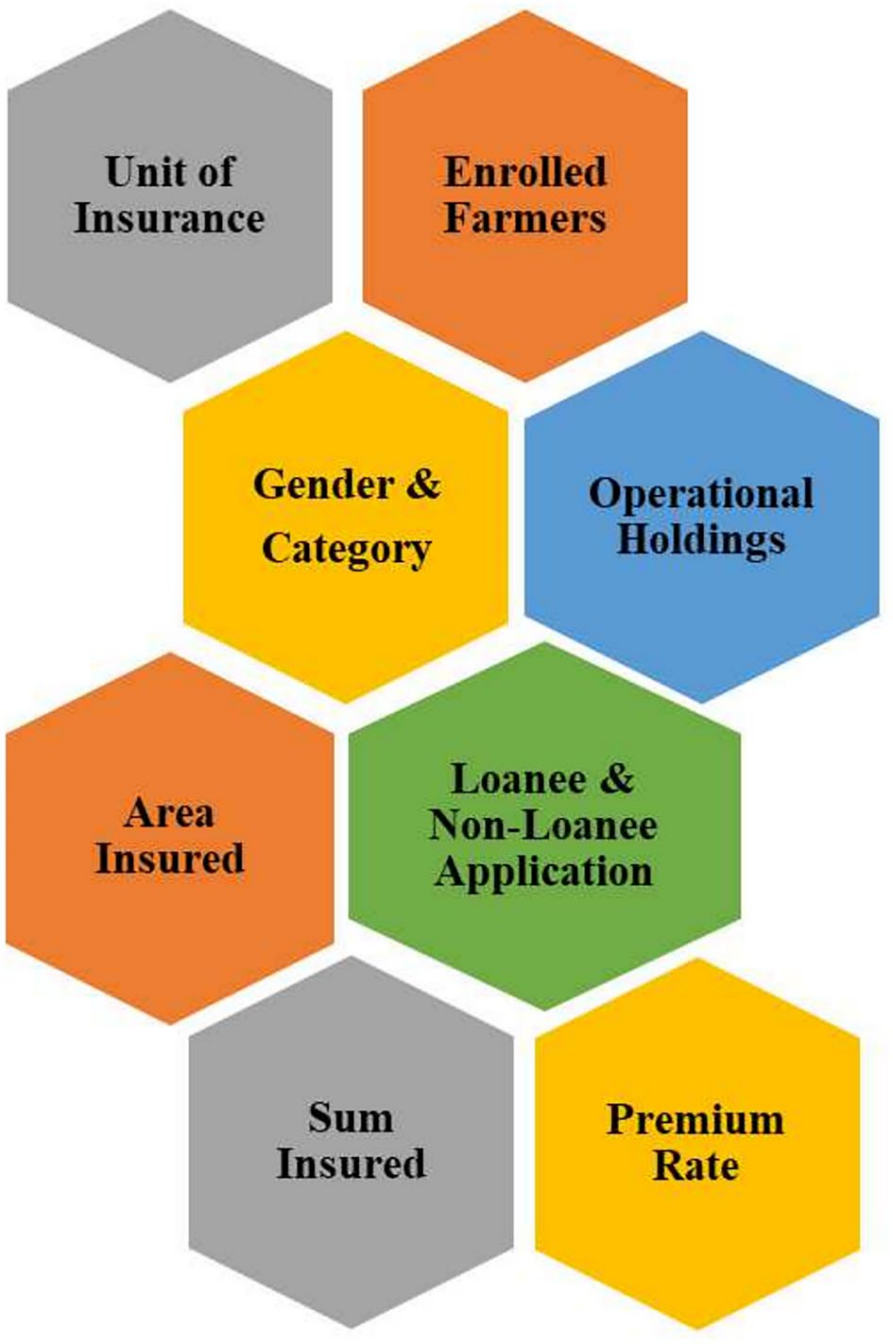
Constraints provided in the PMFBY scheme’s raw dataset.

This research employed spatiotemporal and longitudinal time-series data from various government sources, such as the State Crime Records Bureau (SCRB) and the Agricultural Census of India. The primary data source was the SCRB’s report titled “Crime in Maharashtra,” which contained statistics on farmer suicides, classified by whether the farms were irrigated or unirrigated, at both district and division levels. The study also incorporated information from the 2018 to 2021 PMFBY scheme report, with the constraints mentioned in Fig. 2.

The selection of constraints from the PMFBY dataset was driven by prior literature and theoretical considerations about farmer vulnerability and agrarian distress. Variables such as ‘Area Insured’, ‘Sum Insured’, and ‘Unit of Insurance’ capture the extent and affordability of financial protection offered to farmers. These have been recognized as critical factors influencing farmer resilience, as they determine the degree of economic security in the event of crop failure^24,50^. The Farmer, State, and GOI Premiums reflect government support; higher contributions from these entities are associated with broader coverage and reduced farmer burden, thereby improving scheme accessibility^54,58^. The distinction between Loanee and Non-Loanee farmers is vital, since loanee farmers are auto-enrolled in the scheme while non-loanees must voluntarily opt in, which may leave them underinsured and more vulnerable due to systemic exclusion^55,56^. Demographic indicators such as gender and caste (SC, ST, OBC, General) are included to explore disparities in scheme access and the differential impact of PMFBY across social groups^61^. Prior studies have shown that marginalized communities often face institutional and informational barriers, exacerbating their exposure to agrarian distress^26^. Additionally, landholding size categories (Marginal, Small, Others) are crucial proxies for economic stability. Marginal and small farmers, who typically lack adequate buffers against crop loss, are disproportionately affected by agricultural uncertainties^19^. Rainfall, as an environmental constraint, has been consistently linked to crop outcomes and suicide trends in Indian agriculture^5,13^.

The comprehensive data set containing all the PMFBY constraints and considered independent variables, along with their concise explanations, is provided in Table 1. Furthermore, rainfall data was acquired from the Rainfall Recording and Analysis Department of the Maharashtra Agriculture sector. The PMFBY data was restructured by combining seasonal rabi and kharif crop data for each year, transforming it into an annual dataset to address missing value issues. Similarly, the total number of farmer suicides based on landholding status is utilized for this study to facilitate analysis.

**Table 1:** Overview of considered independent variable for logistic regression modelling.

PMFBY Constraints	Variables	Description
Unit of insurance		‘Defined areas’ for each notified crop for widespread calamities
Farmers		All farmers growing notified crops in a notified area during the season who have insurable interest
Application	Loanee	Farmers who have been sanctioned for Short-term seasonal Agricultural Operations (SAO) loans/Kisan Credit Card (KCC) for the notified crops from defined FIs
	Non-loanee	Farmers who have not availed of any agricultural loan from any recognized financial institution
Area insured (thousand hect.)		It is the gross cropped area as per the unit of insurance sanctioned in a notified area
Premium (amount in lac.)	Farmer	The amount payable by the farmers (with the premium rate for most crops being just 2% for Kharif crops and 1.5% for Rabi crops)
	State	The amount payable by the state government
	GOI	The amount payable by the Government of India
Sum insured (in lac.)		The value of the crop at the time of sowing. The amount is fixed based on the area and crop type
Gender($\%$)	Male	Percentage of male farmers enrolled under the scheme PMFBY
	Female	Percentage of female farmers enrolled under the scheme PMFBY
	G_Others	Percentage of other farmers enrolled under the scheme PMFBY
Category ($\%$)	SC	Percentage of scheduled caste farmers enrolled under the scheme PMFBY
	ST	Percentage of scheduled caste farmers enrolled under the scheme PMFBY
	General	Percentage of general category farmers enrolled under the scheme PMFBY
	OBC	Percentage of other backward classes farmers enrolled under PMFBY
Operational holding ($\%$)	Marginal	Cultivator with a land holding of 1 hectare or less (2.5 acres)
	Small	Cultivator with a land holding of 2 hectares (5 acres) or less, as defined in the land ceiling legislation of the concerned State/ UT
	F_Others	A farmer cultivating, as owner or tenant or sharecropper, agricultural land of more than 2 hectares (more than 5 acres)
Annual rainfall		Average annual rainfall for the districts of Maharashtra

### Spatiotemporal approach

Let $R$ represent the entire region and $S$ denote the total number of suicide instances within this area. The moving circular window identifies a set of windows, referred to as $w$. Each window corresponds to a potential high-risk region (cluster) surrounded by the centroids representing the census districts of Maharashtra.

The null hypothesis typically asserts that events are randomly distributed across space and time, following a homogeneous Poisson process, while the alternative hypothesis suggests that there is variation in the distribution of events across space and time. In the analysis, $s(w)$ represents the actual number of suicide instances within each window, which is compared to the expected instances, $\mu (w)$. When a window shows $s(w)$ greater than $\mu (w)$, it indicates the presence of a cluster. Subsequently, the likelihood of observing a higher number of events in the cluster can be compared to the likelihood of observing the same number of events under the null hypothesis. The likelihood function for the alternative hypothesis can be expressed as follows:

1 $$\begin{aligned} L(w) =\ sup\left( \frac{s(w)}{\mu (w)}\right) ^{s(w)}\left( \frac{S-s(w)}{S-\mu (w)}\right) ^{S-s(w)}I\left( \frac{s(w)}{\mu (w)}>\frac{S-s}{S-\mu (w)}\right) . \end{aligned}$$

In this context, *I*() represents an indicator variable that takes a value of 1 when the window has more cases than expected under the null hypothesis, and otherwise, it takes a value of 0.

The null hypothesis assumes that the anticipated instances within any possible cluster are proportional to the population size of that cluster^62^. Indirect standardization and covariate adjustment can be applied to estimate the expected instances within each window^63^.

**Fig. 3:**
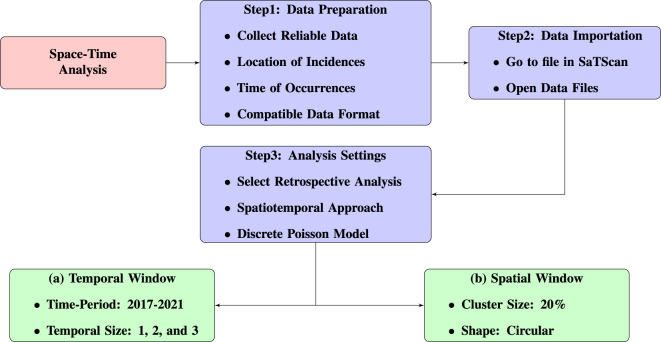
Spatiotemporal analysis workflow of the study.

Suppose $c_i$ is the actual instances in the jth covariate category for each window, then $d_i$, $C_i$, and $D_i$ denote the respective population size, actual instances, and jth covariate overall population within the spatial region R, the adjusted $\mu (w)$ is calculated as follows:

2 $$\begin{aligned} \mu (w)\ =\ \sum _{j}{E\left( c_j\right) =\sum _{j}\frac{d_j\ c_j}{D_j}}. \end{aligned}$$

To assess the statistical significance of the obtained cluster, we use a statistical test that compares what’s observed to what’s expected under normal conditions. If the observed numbers are much higher than expected, it suggests a real pattern, not just random chance. This comparison, called a likelihood ratio, helps us identify meaningful clusters and guide targeted interventions. Subsequently, Monte Carlo testing is conducted by simulating 999 replications of the dataset. For each simulated dataset, the likelihood ratio for the most likely cluster is calculated using the same approach as applied to the actual data^64^. To evaluate whether the observed number of events in the most likely cluster significantly deviates from the expected count, the rank of the maximum likelihood function from the real data is compared to the likelihood ratios obtained from the simulated datasets^65^. Thus

3 $$\begin{aligned} p-value\ =\ \frac{\gamma }{\left( 1+ S\right) }, \end{aligned}$$

where $\gamma$ is the rank of the maximum likelihood ratio derived from the actual dataset, while S indicates the number of simulations carried out.

A detailed explanation of the entire methodology and the steps involved in the spatiotemporal analysis are provided through the workflow mentioned in Fig. 3.

This method directly supports research question-1 by enabling the detection of persistent spatiotemporal patterns indicative of elevated suicide risk. By applying varying temporal window sizes, this methodology captures both short and long duration clusters, thus accounting for temporal dynamics in suicide prevalence. The outputs of this phase, including relative risk (RR) and log-likelihood ratios (LLR), serve as the foundation for subsequent regression modelling. This analysis informs research question-2 by classifying districts into clustered and non-clustered categories, thereby facilitating a comparative assessment of socio-demographic and scheme-specific attributes between high-risk and low-risk regions. Further, a binary logistic regression model was constructed to estimate the likelihood of a district belonging to a suicide cluster (clustered = 1, non-clustered = 0) as a function of multiple predictor variables drawn from PMFBY and related socio-economic indicators. This analytical component addresses research question-3, which seeks to evaluate the differential influence of these factors before and after the PMFBY’s implementation.

### Logistic regression (LR) approach

LR models are among the most commonly used models within the generalized linear models (GLM) framework. A logistic regression model is applied when the nature of the response variable *y* is dichotomous. The core idea of a regression model is to connect the mean response to a set of predictors. When the response variable *y* is binary, the mean response is expressed as $E(y) = P(y=1)$, a value between 0 and 1, while the predictors can take real values. Therefore, a link function is required to ensure consistency in the data ranges on both sides.

**Fig. 4:**
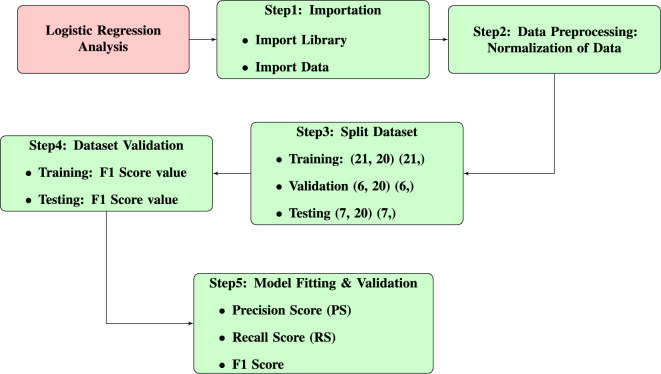
Logistic regression modelling workflow of the study.

At its core, logistic regression helps us understand the likelihood of an event, like whether a region falls into a high-risk suicide cluster, based on various influencing factors. To do this, it uses a concept called the logit, which transforms probabilities into a scale suitable for analysis. In simpler terms, it shows how changes in a particular factor increase or decrease the odds of suicide risk in an area. It is the natural logarithm of an odds ratio, that is,

4 $$\begin{aligned} G(\mu )=log\left( \frac{\mu }{1-\mu }\right) .\ \ \end{aligned}$$

Here, $\mu = E(y) = P(y=1)$, and with the function $G(\mu )$, it can take any real value. The corresponding generalized linear model (GLM) is expressed as the following logistic regression model:

5 $$\begin{aligned} log\left( \frac{\mu _i}{1-\mu _i}\right) \ =\ \beta _1x_{i1}+\beta _2x_{i2}+\cdots +\beta _kx_{ik}, \qquad \qquad i= 1,2,...n. \end{aligned}$$

Here, $\mu _i = E(y_i) = P(y_i=1)$, and $y_i$ is assumed to follow a binomial distribution with a mean of $\mu _i$.

Estimates of the $\beta 's$ can be derived using the maximum likelihood method. The likelihood function *L* is the joint probability distribution evaluated at the observed values $y_i$. Therefore,

6 $$\begin{aligned} L(\beta _1, \beta _2.....,\beta _k)\ =\ \prod _{j=1}^{n}\left( P\left( \mu _i\right) \right) ^{y_i}\left( 1-\ P\left( \mu _i\right) \right) ^{1-y_i}. \end{aligned}$$

A key benefit of the logistic regression model is its straightforward interpretation: $\log (\mu _i/(1-\mu _i))$ can be understood as the odds of the event “$y_i=1$”. Consequently, the parameter $\beta _k$ can be interpreted as the change in odds on a logarithmic scale when the predictor $x_i$ is increased by 1 unit^66^.

The detailed workflow of the adopted methodology and the steps involved in the logistic regression analysis are provided through Fig. 4.

#### Key evaluation metric

After fitting a model, we validated the model using key matrices such as accuracy score, PS, RS, and F1 score, which are briefly described and formulated in Table 2.

**Table 2:** Evaluation criteria for logistic regression model.

Key metric	Description	Mathematical formulation
Accuracy	The overall predicted accuracy of the model	$\frac{{TP}+{TN}}{{TP}+{TN}+{FP}+{FN}}$
Precision score (PS)	Indicates how many values, out of all the predicted positive values, are actually positive	$\frac{{TP}}{{TP}+{FP}}$
Recall score (RS)	Measures how good our model is at correctly predicting positive classes	$\frac{{TP}}{{TP}+{FN}}$
F1-score	Harmonic mean of precision and recall	$$\frac{2*PS*RS}{PS+RS}$$

*TP* true positives, *FP* false positive, *TN* true negative, *FN* false negative, *PS* precision score, *RS* recall score.

## Results

### Exploratory analysis

**Fig. 5:**
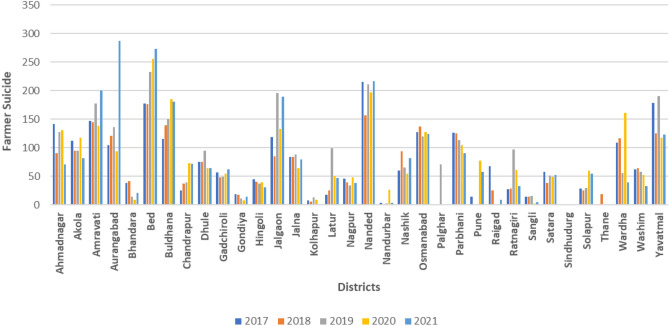
Distribution of farmers’ suicides from 2017 to 2021 (Created by Microsoft Excel, Version 2411, https://www.microsoft.com/en-in/microsoft-365/excel).

**Fig. 6:**
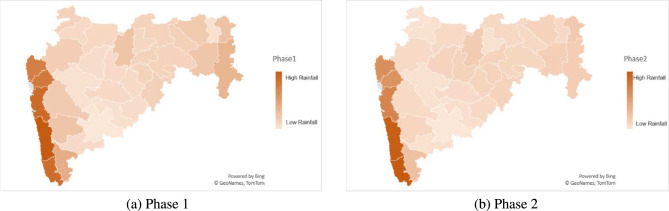
Phase-wise rainfall distribution (Created by Microsoft Excel, Version 2411, https://www.microsoft.com/en-in/microsoft-365/excel).

**Fig. 7:**
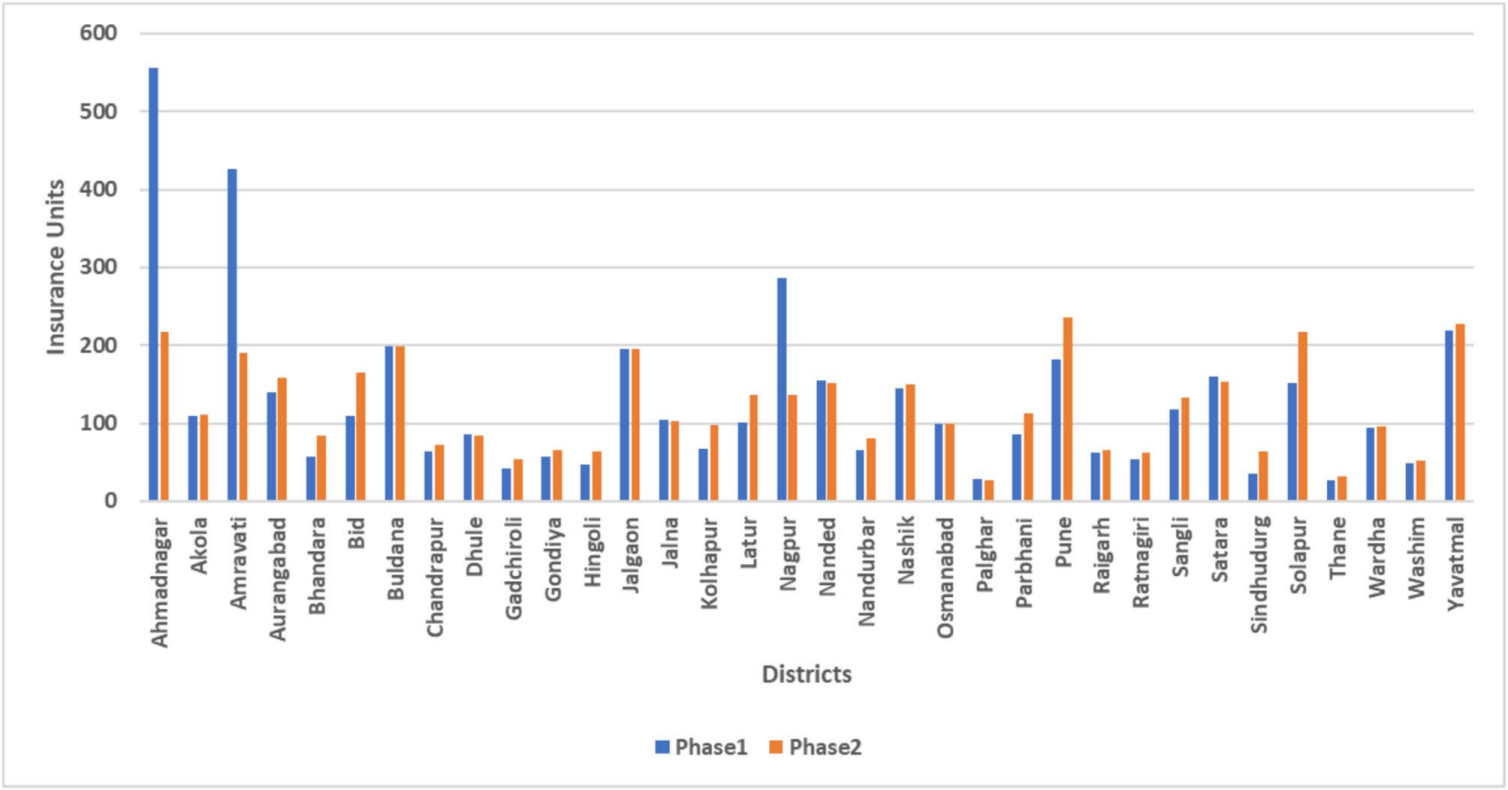
Phase-wise insurance units distribution (Created by Microsoft Excel, Version 2411, https://www.microsoft.com/en-in/microsoft-365/excel).

The distribution of suicidal mortality for the time frame 2017–2021 across the 34 districts of Maharashtra is illustrated in Fig. 5. In the most recent year, 2021, the district of Aurangabad had the highest number of suicide cases, followed by Buldhana, Amravati, and Nanded. Additionally, Fig. 6 displays the distribution of annual rainfall across these districts in different phases, showing a similar trend in rainfall among the districts in each phase. Furthermore, Fig. 7 presents the allocation of insurance units by the PMFBY scheme on a district-wide basis. In Phase 1, the district of Ahmadnagar received the highest number of units, followed by Amravati, Nagpur, and Yavatmal. Similarly, in Phase 2, Pune, Solapur, Yavatmal, and Ahmadnagar districts received the maximum number of insurance units.

In the preliminary analysis, we examined the correlation between suicide mortality and all the independent variables listed in Table 1, with specific reference made in Table 3. In the non-parametric Spearman’s correlation analysis during Phase 1, the results indicated a significant and positive association between suicide mortality and the following variables: Farmer Premium (0.735), Farmers (0.677), and Area Insured (0.658). Conversely, there was a negative correlation with Rainfall (− 0.373), Male (− 0.337), and Marginal (− 0.325). Similarly, during Phase 2, Spearman’s correlation coefficients revealed a significant and positive relationship between suicide mortality and the variables Farmer Premium (0.680), Females (0.618), and Sum Insured (0.600). On the other hand, there was a negative correlation with Male (− 0.618), Rainfall (− 0.389), and Marginal (− 0.256).

Additionally, Tables 4 and 5 offer phase-specific descriptive statistics. Descriptive statistics encompass a set of techniques used to summarize and elucidate the key attributes of a dataset. These statistical measures provide a succinct and lucid overview of the data, facilitating its interpretation and comprehension. They offer insights into various aspects of the data, such as the central tendency, data dispersion around the mean, skewness of the data distribution, and the distribution’s peakedness or flatness for each variable. In Phase 1, certain variables, such as ST (with values 24.092 and 4.663), SC (with values 17.935 and 4.072), and Insurance Units (with values 6.782 and 2.403), exhibit a more peaked distribution and a longer tail on the right side. In contrast, General (with values − 1.482 and − 0.256), OBC (with values − 1.176 and 0.441), and Female (with values − 1.061 and − 0.161) indicate a flatter distribution with a longer tail on the left side. Turning to Phase 2’s descriptive statistics, variables like Other (with values 25.904 and 4.909), ST (with values 12.079 and 3.383), and Marginal (with values 5.030 and 1.908) suggest a heavy-tailed distribution with a longer tail on the right. Conversely, General (with values − 1.452 and − 0.176), OBC (with values − 1.321 and 0.309), and Insurance Units (with values − 0.915 and 0.415) present a light-tailed distribution with a longer tail on the left.

**Table 3:** Phase-wise correlation between suicide instances and other independent variables.

Constraints	Phase1	Phase2
Suicide	1	1
Farmer premium	0.735	0.680
Farmers	0.677	0.521
Area insured	0.658	0.583
Sum insured	0.649	0.600
Non-loanee	0.644	0.547
State premium	0.631	0.591
GOI premium	0.631	0.591
Insurance	0.582	0.421
Small	0.362	0.320
Female	0.340	0.618
General	0.136	0.185
Loanee	0.118	0.435
SC	− 0.023	− 0.197
OBC	− 0.054	− 0.033
G_Others	− 0.232	− 0.087
F_Others	− 0.239	− 0.218
ST	− 0.291	− 0.207
Marginal	− 0.325	− 0.256
Male	− 0.337	− 0.618
Rainfall	− 0.373	− 0.389

**Table 4:** Descriptive statistics for the phase1.

Variables	Mean	Standard error	Standard deviation	Kurtosis	Skewness
Insurance	128.706	19.110	111.428	6.782	2.403
Farmers	103,364.882	23,059.666	134,459.800	3.734	1.946
Loanee	27,412.221	4421.892	25,783.840	3.118	1.645
Non-loanee	221,249.221	62,645.129	365,280.733	5.662	2.279
Area insured	137.889	29.297	170.828	2.057	1.600
Farmer premium	1059.596	218.103	1271.746	3.718	1.837
State premium	3811.921	1035.856	6040.024	4.320	2.177
GOI premium	3811.921	1035.856	6040.024	4.320	2.177
Sum insured	45,172.258	9822.046	57,271.878	3.161	1.837
Male	84.558	0.688	4.010	− 1.069	0.159
Female	15.397	0.689	4.015	− 1.0612	− 0.161
G_Others	0.045	0.007	0.043	5.751	2.178
SC	5.172	1.932	11.268	17.935	4.072
ST	5.941	1.743	10.165	24.092	4.663
OBC	40.386	4.305	25.103	− 1.176	0.441
General	48.501	4.793	27.949	− 1.482	− 0.256
Marginal	13.370	1.321	7.702	3.999	1.750
Small	71.489	2.559	14.923	1.582	− 1.330
F_Others	15.140	2.212	12.898	3.375	1.810
Rainfall	1131.424	164.991	962.054	1.964	1.775

**Table 5:** Descriptive statistics for the phase2.

Variables	Mean	Standard error	Standard deviation	Kurtosis	Skewness
Insurance	120.324	10.227	59.634	− 0.915	0.415
Farmers	67,593.492	13,124.636	76,529.125	1.396	1.425
Loanee	20,175.184	3741.506	21,816.543	1.699	1.593
Non-loanee	140,243.669	36,404.453	212,272.616	4.483	2.063
Area	90.468	17.821	103.912	1.029	1.341
Farmer premium	787.377	149.351	870.860	0.685	1.216
State premium	3836.158	1039.419	6060.802	3.586	2.081
GOI premium	3675.831	975.095	5685.733	3.148	2.005
Sum	34,465.005	7042.269	41,063.134	1.431	1.483
Male	83.951	0.827	4.820	0.593	− 0.456
Female	16.011	0.829	4.831	0.570	0.459
G_Others	0.039	0.013	0.076	25.904	4.909
SC	4.027	0.682	3.977	4.713	2.079
ST	4.432	1.446	8.431	12.079	3.383
OBC	42.371	4.424	25.797	− 1.321	0.309
General	49.170	4.950	28.863	− 1.452	− 0.176
Marginal	11.930	0.948	5.525	5.030	1.908
Small	73.167	1.864	10.866	0.055	− 0.455
F_Others	14.903	1.575	9.184	0.202	0.762
Rainfall	1227.985	150.366	876.778	4.074	2.216

### Spatiotemporal analysis

As Phase1 and Phase2 spanned a maximum duration of 3 years, the analysis was conducted by setting the temporal window parameter to a minimum of 1 and 2 year and a maximum of 3 years. This configuration aimed to identify both short-term and long-term patterns of persistent clusters. Additionally, utilizing multiple temporal windows helps ensure that clusters are not overlooked due to a single, fixed window size. This approach enhances the analysis’s reliability and robustness by validating results across various time scales. For each of these temporal window values, circular window shapes were chosen due to their effectiveness in detecting smaller, centralized clusters. Circular shapes are particularly well-suited for scenarios where clusters are compact and symmetric, as they offer precise and focused detection. This configuration enhances the reliability of identifying spatial patterns within the given temporal windows. To ensure the accuracy and realism of detected clusters, the maximum spatial cluster size was restricted to 20% of the at-risk population. This limitation prevents the inclusion of neighboring areas that may not genuinely contribute to the cluster, avoiding the overestimation of cluster size. By doing so, the analysis remains focused on regions with a significant concentration of incidents, ensuring more precise localization of clusters. The discrete Poisson probability model was employed for scanning due to the relatively low occurrence of suicides. The window exhibiting the highest likelihood is designated as the primary cluster area, while other clusters with statistically significant log-likelihood ratios (LLR) are termed secondary potential clusters. The p-values for LLR were determined using 999 Monte Carlo simulations. A p-value less than 0.05 signifies a significantly elevated risk within the scanning window, suggesting a potential cluster with high suicide risk. To assess the risk in suicide cluster areas, the relative risk (RR) of suicide was computed for each cluster.

**Fig. 8:**
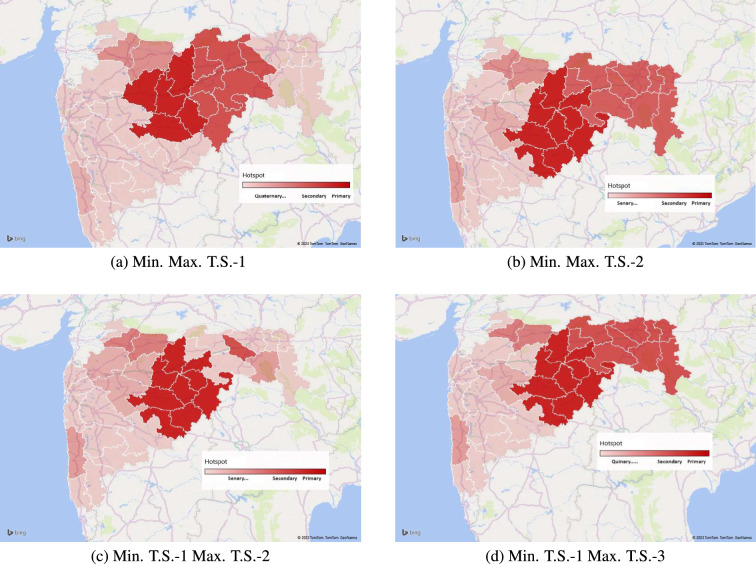
Suicide cluster of different temporal window sizes under consideration (Created by Microsoft Excel, Version 2411, https://www.microsoft.com/en-in/microsoft-365/excel).

We have listed down all significant clusters ($p-value < 0.05$) in Table 6 that were found from each category of window temporal size in the district cluster analysis. The visualization of all the clusters is provided through the choropleth graph in Fig. 8. Upon examining the visualization and referring to the Table 6, it becomes evident that, in the year 2017, prior to the implementation of PMFBY, the districts of Yavatmal, Wardha, Washim, Amravati, Hingoli, Akola, and Nanded emerged as the primary cluster with high relative risk and lower p-value for farmer suicides, with a temporal window size of 1. Moving forward to the year 2019, one year after the program’s implementation, Dhule and Jalgaon (both in the Nashik division), as well as Ratnagiri (in the Konkan Division), were identified as the prominent areas with a high incidence of farmer suicides. In 2021, the farmer suicide cluster shifted to the districts of the Aurangabad division, specifically Jalna, Beed, Parbhani, Aurangabad, and one district from the Amravati division, which is Buldhana.

In the early stages of implementing PMFBY with a temporal window size of 2 and considering Min. T.S.-1 and Max. T.S.-2, Gondiya, Bhandara, Nagpur, Chandrapur, Wardha, Gadchiroli, Yavatmal, Amravati, and Akola districts are identified as significant areas with a high incidence of suicide. On the flip side, for the years 2020 and 2021, Wardha, Ahmednagar, Nashik, and Aurangabad districts are noted as major suicide clusters.

**Fig. 9:**
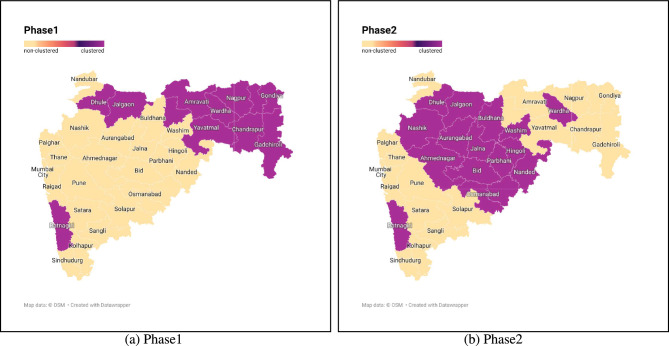
Phase-wise farmer’s suicide cluster in the state of Maharashtra, India (Created by Map data: $\copyright$ OSM Datawrapper, https://www.datawrapper.de/).

As we transition to the temporal window range of Min. T.S.-1 and Max. T.S.-3, it becomes evident that during the initial period (2017-2019) of the implementation, the regions of Gondiya, Bhandara, Nagpur, Chandrapur, Wardha, Gadchiroli, Yavatmal, Amravati, and Akola predominantly exhibit higher instances of farmer suicides. Similarly, in the subsequent working phase of the PMFBY scheme, Parbhani, Hingoli, Jalna, Nanded, Latur, Beed, Washim, Osmanabad, Buldana, Dhule, Jalgaon, and Ratnagiri districts come to the forefront as emerging clusters for such incidents. The specific outcomes for each phase within the Min. T.S.-1 and Max. T.S.-3 temporal windows are presented in Table 7 and visually represented in Fig. 9.

**Table 6:** Significant suicide cluster obtained at the different temporal window sizes.

Time-frame	(a) Min. Max. T.S.-1					
	Cluster	N($\omega$)	$\mu (\omega )$	RR	LLR	p-value
2021	Jalna, Beed, Parbhani, Aurangabad, Buldana	912	310.21	3.09	396.72	< 0.0000001
2017	Yavatmal, Wardha, Washim, Amravati, Hingoli, Akola, Nanded	868	355.36	2.55	273.45	< 0.0000001
2019	Dhule, Jalgaon	291	153.81	1.91	49.12	< 0.0000001
2019	Ratnagiri	97	39.55	2.46	29.70	< 0.0000001

	(b) Min. Max. T.S.-2					
2019–2020	Parbhani, Hingoli, Jalna, Nanded, Latur, Beed, Washim, Osmanabad, Buldana	2185	922.72	2.66	692.34	< 0.0000001
2017–2018	Gondiya, Bhandara, Nagpur, Chandrapur, Wardha, Gadchiroli, Yavatmal, Amravati, Akola	1397	941.79	1.54	104.66	< 0.0000001
2019–2020	Dhule, Jalgaon	489	308.04	1.61	46.37	< 0.0000001
2019–2020	Ratnagiri	158	79.21	2.01	30.56	0.000000000011

	(c) Min. T.S.-1 and Max. T.S.-2					
2019–2020	Parbhani, Hingoli, Jalna, Nanded, Latur, Beed, Washim, Osmanabad, Buldana	2185	922.72	2.66	692.34	< 0.0000001
2020	Wardha	161	31.94	5.09	132.03	< 0.0000001
2019	Dhule, Jalgaon	291	153.81	1.91	49.12	< 0.0000001
2019–2020	Ratnagiri	158	79.21	2.01	30.56	0.000000000028
2021	Ahmednagar, Nashik, Aurangabad	440	351.45	1.26	10.64	0.0027

	(d) Min. T.S.-1 and Max. T.S.-3					
2019–2021	Parbhani, Hingoli, Jalna, Nanded, Latur, Beed, Washim, Osmanabad, Buldana	3262	1383.45	2.83	1086.95	< 0.0000001
2017–2019	Gondiya, Bhandara, Nagpur, Chandrapur, Wardha, Gadchiroli, Yavatmal, Amravati, Akola	2065	1412.68	1.55	151.10	< 0.0000001
2019–2021	Dhule, Jalgaon	742	461.84	1.64	74.91	< 0.0000001
2019–2020	Ratnagiri	158	79.21	2.01	30.56	0.000000000050

**Table 7:** Detailed description of regions obtained as the farmers’ suicide cluster in the specific phases.

Phase	Significant clusters	Division
Phase1	Bhandara, Chandrapur, Gadchiroli, Gondiya, Nagpur, Wardha	Nagpur
	Amravati, Akola, Yavatmal	Amaravati
	Dhule, Jalgaon	Nashik
	Ratnagiri	Konkan
Phase2	Aurangabad, Beed, Hingoli, Jalna, Latur, Nanded, Osmanabad, Parbhani	Aurangabad
	Ahmednagar, Dhule, Jalgaon, Nashik	Nashik
	Buldana, Washim	Amaravati
	Ratnagiri	Konkan
	Wardha	Nagpur

### Logistic regression analysis

This research employed Python software to conduct logistic regression modeling. To ensure the robustness and reliability of the logistic regression model, we initially conducted a multicollinearity assessment among the PMFBY constraints. Variance Inflation Factor (VIF) was calculated for each variable to detect potential multicollinearity issues. The analysis revealed that certain variables, specifically F_Others under “Operational Holding”, G_Others under “Gender”, GEN under “Category”, and S_Premium under “Premium”, exhibited high multicollinearity. These variables were also contributing to inflated VIF values within their respective groups. To mitigate this issue and enhance model stability, we excluded these variables from the modelling. The revised VIF values, indicating a significant reduction in multicollinearity following the exclusion of these variables, are summarized in Table 8. The VIF value indicates that the majority of variables exhibit values below the threshold of 10, which is considered acceptable in multicollinearity diagnostics. This suggests that these variables are appropriate for inclusion in the logistic regression model and do not pose a threat to the model’s validity.

**Table 8:** Phasewise variance inflation factor (VIF) for each variable.

Variables	VIF	
	Phase1	Phase2
Female	1.989	1.237
Male	1.981	1.226
Sum	5.632	3.943
Area	1.703	3.378
GOI_Premium	8.802	9.573
Farmers	7.039	8.537
F_Premium	6.521	4.767
NonLonee	4.758	3.968
Small	5.381	4.694
Lonee	4.091	2.87
Marginal	3.886	3.465
Rainfall	2.919	3.888
OBC	2.635	1.838
ST	2.539	1.327
Insurance	2.012	2.709
SC	1.551	1.526

Logistic regression analysis seeks to comprehend how the model predicts the likelihood of an event happening based on predictor variable values. Given that we have acquired data on suicide occurrences in both clustered and non-clustered areas in each phase, we utilized logistic regression to discern the factors influencing farmer suicides covered by the PMFBY in the clustered regions (indicated as 1) in comparison to the non-clustered regions (indicated as 0).

**Table 9:** Phase-wise logistic regression model validation.

Phases	Key metrices			
	Cluster	Precision	Recall	F1 score
Phase1	0 (No)	1	0.80	0.89
	1 (Yes)	0.67	1	0.80
Phase2	0 (No)	0.83	1	0.91
	1 (Yes)	1	0.50	0.67

During pre-processing, the data was examined for null values, but none were found in the restructured PMFBY dataset. Next, normalization was performed using the MinMaxScaler function to standardize the data. Subsequently, the dataset was divided into training and testing sets with a 70/30 split. These steps represent a standard procedure for each phase of analysis. The count values for the non-clustered region in phase1 were 0.619, 0.667, and 0.714, while for the clustered region, they were 0.381, 0.333, and 0.286 for the training, validation, and testing datasets, respectively. Similarly, in phase2, the count values for the non-clustered region were 0.523, 0.667, and 0.714, and for the clustered region, they were 0.476, 0.333, and 0.286 for the training, validation, and testing datasets, respectively. Subsequently, we computed the F1 score for the partitioned dataset, which proved to be highly significant, reaching approximately 85% and 86% for the training and testing datasets in both phase1 and phase2, respectively.

**Fig. 10:**
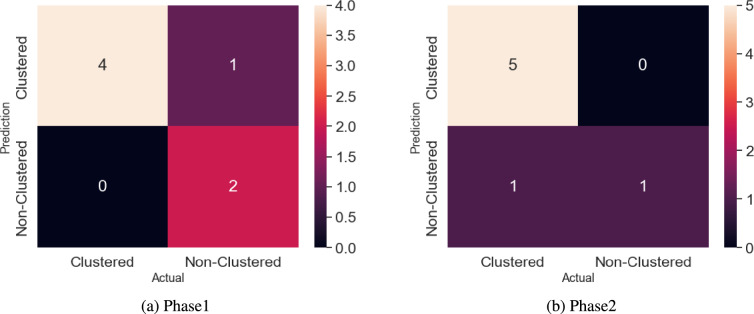
Phase-wise confusion matrix of clustered and non-clustered regions (Created by Python 3.10.9, https://www.python.org).

After validating the dataset, we assessed the performance of the logistic regression model, resulting in accuracy scores of 86% for both phase1 and phase2 models. Moreover, the positive outcomes of the fitted models in each phase were corroborated by the confusion matrix and key metrics. Figure 10 presents the obtained confusion matrix, while Table 9 displays the key metrics for each model. Table 9 highlights F1 Score values of 0.89 and 0.80 for non-clustered and clustered regions in phase1, and similarly, phase2 demonstrates F1 Score values of 0.91 and 0.67 for the non-clustered and clustered regions. Additionally, the overall precision values for the phase1 model range from 0.83 to 0.90, considering both macro and weighted averages. Similarly, the overall recall values range from 0.90 to 0.86 in phase1. For phase2, the precision values range from 0.92 to 0.88 when assessing macro and weighted averages, and the recall values range from 0.75 to 0.86. In a similar manner, the F1 Score for macro and weighted averages in the phase1 model stands at 0.84 and 0.86, while the phase2 model attains F1 Score values of 0.79 and 0.84 for the macro and weighted averages, respectively.

**Fig. 11:**
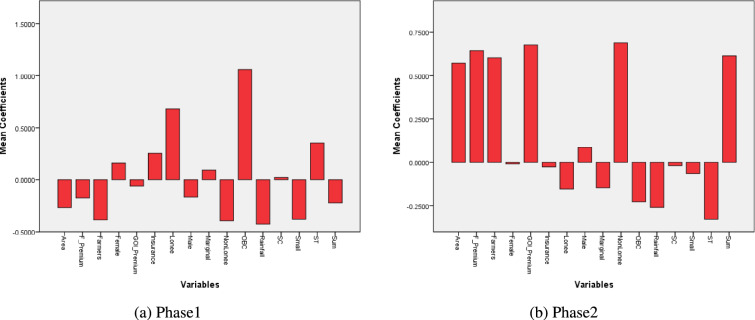
Phase-wise variable coefficients of logistic regression modeling (Created by Python 3.10.9, https://www.python.org).

A detailed representation of how the coefficients of the variables provided by the PMFBY scheme impact the clustered region is visualized in Fig. 11 for each phase.

## Discussion

The software SaTScan is employed to perform spatiotemporal cluster detection and assess the significance of these clusters. During Phase 1, farmer suicide clusters were predominantly located in the districts of Bhandara, Chandrapur, Gadchiroli, Gondiya, Nagpur, and Wardha within the Nagpur division, as well as Amravati, Akola, and Yavatmal in the Amravati division. These regions have historically faced lower rainfall, poor irrigation infrastructure, and a high reliance on risk-prone crop patterns. The agrarian population in these areas largely comprises small and marginal farmers with limited access to institutional credit, heightening their financial vulnerability. Furthermore, the implementation of the Pradhan Mantri Fasal Bima Yojana (PMFBY) during this period was still in its initial stages, with uneven coverage and limited protective efficacy. In Phase 2, the clustering of suicides shifted to the districts of Aurangabad, Beed, Hingoli, Jalna, Latur, Nanded, Osmanabad, and Parbhani in the Aurangabad division, as well as Ahmednagar, Dhule, Jalgaon, and Nashik in the Nashik division. This spatial transition can be attributed to multiple socio-economic and climatic factors. Improved implementation of PMFBY in the earlier hotspot regions may have contributed to a reduction in suicides there. Meanwhile, newly emerging clusters corresponded with localized crop failures, moderate rainfall deficits, and possible gaps in awareness, accessibility, or claim settlement processes related to PMFBY, thereby delaying its impact in these areas.

The regression model analysis indicates that, in Phase 1, key factors positively associated with the formation of suicide clusters included insurance units, loanee status, belonging to the Other Backward Class (OBC) category, and individuals identifying under the “marginal” operational holdings. In Phase 2, significant contributors to clustering were identified as the area insured, number of farmers, farmer premium, Government of India (GOI) premium, non-loanee status, and sum insured. Conversely, certain variables functioned as constraints, reducing the likelihood of cluster formation. In Phase 1, these included the Farmer and GOI premium, area insured, non-loanee status, small landholding status, and rainfall. In Phase 2, constraining factors included loanee status, marginal category, Scheduled Tribe (ST) category, OBC category, unit of insurance, and rainfall. Rainfall, in particular, demonstrated a negative correlation with suicide mortality across both phases, underscoring its critical role in agrarian distress. Variability in rainfall directly impacts crop yields, especially in rain-fed agricultural regions. Although PMFBY is intended to mitigate such shocks through financial compensation, its effectiveness is contingent upon timely and sufficient implementation. The logistic regression results further reveal that variables such as area insured, sum insured, and premium subsidies provided by state and central governments had a significant mitigating effect in districts with higher PMFBY coverage. However, in regions experiencing persistent rainfall deficits coupled with limited scheme penetration or delayed disbursements, the scheme’s capacity to alleviate distress was markedly reduced, as evidenced by continued high suicide rates.

The analysis results indicate that the constraints of the PMFBY scheme, particularly area insured, sum insured, and premiums, play a crucial role in significantly reducing farmer suicides in clustered areas after the scheme’s implementation. This positive impact is more pronounced when compared to non-clustered regions. The structured allocation of insurance units and the affordability of premiums in clustered areas likely enhance farmers’ access to financial protection against crop losses, reducing economic stress and contributing to a decline in suicide rates. This highlights the importance of targeted strategies and localized implementation of PMFBY to maximize its effectiveness in safeguarding farmers’ livelihoods^67^.

While this study highlights the role of PMFBY in reducing suicide rates in clustered regions, it also identifies constraints such as low coverage in certain districts and disparities in benefit allocation. These findings are consistent with prior critiques of the scheme, studied by Cariappa et al. 51 and Tiwari et al. 54, noted that PMFBY’s penetration was uneven across regions, with Western states exhibiting better implementation (45.4%) compared to North-Eastern regions (only 3.13%). Similarly, Roy et al. 6 emphasized issues related to delayed claim settlements and lack of awareness among farmers in remote areas, which echoes the constraints observed in districts like Wardha and Ratnagiri in this study during Phase 2. Moreover, previous evaluations by Bhushan and Kumar 24 and Shrimali et al. 59 underscored administrative inefficiencies and the need for region-specific targeting, a point reinforced by our findings, where localized spatiotemporal clusters highlighted the importance of tailored interventions.

Since this study identifies significant spatiotemporal clusters of farmer suicides in Maharashtra and the mitigating role of PMFBY, it is important to situate these findings within a broader socio-political and environmental context. Similar patterns of agrarian distress have been observed in other Indian states such as Karnataka, Andhra Pradesh, and Telangana regions that also adopted PMFBY but with varying degrees of implementation success^16^. The opted spatiotemporal logistic regression framework used in this study is generalizable and could be applied across different Indian states to identify region-specific risk patterns and policy responsiveness. Additionally, while policy factors are central to the analysis, non-policy determinants, such as psychological stress, cultural stigma around debt, intergenerational pressure, and the erosion of traditional support systems, may also contribute significantly to suicide clusters. Future research could incorporate qualitative dimensions, such as mental health indicators or interviews with affected families, to enrich the statistical findings. By integrating these multidimensional aspects, the study’s framework can more holistically inform targeted interventions, from financial reforms to psychosocial support systems, necessary to address the root causes of farmer suicides.

## Intervention implications

The findings of this study have important implications for suicide prevention efforts for the farmers of Maharashtra. Several specific steps that can be taken to address this issue are mentioned below:

- *Policy recommendations based on spatiotemporal clusters* The findings from Phase 2 highlight new suicide clusters in districts like Aurangabad, Beed, Jalna, and Parbhani, suggesting a geographical shift in agrarian distress. We recommend that policymakers prioritize these Phase 2 cluster regions for intensified implementation of PMFBY through increased insurance awareness campaigns and allocation of additional government subsidies for premium support in districts with high suicide risk but low insurance penetration.
- *Addressing PMFBY constraint gaps* The regression analysis indicates that PMFBY constraints such as limited area insured, insufficient sum insured, and lower premiums paid are key predictors of persistent distress. Therefore, we recommend implementing differentiated premium structures based on cluster risk level, mandating a minimum coverage threshold per farmer, particularly in marginal and small landholding categories, and enhancing data monitoring at the district level to assess scheme performance and adjust resource distribution dynamically.
- *Improving access to mental health services* The government and allied agencies can collaborate to provide accessible and affordable mental health services to farmers in need. This can help identify and treat mental health issues before they escalate to suicidal tendencies. These interventions should function in parallel with PMFBY to create a dual safety net, protecting both the economic and psychological well-being of farmers.
- *Promoting financial literacy and education* Farmers need to be better informed about their financial options, including loan and insurance schemes. Providing education about financial management and literacy can help farmers make informed decisions and reduce financial stress.
- *Developing region-specific interventions* As every region has different climatic and demographic conditions, developing region-specific interventions, including crop diversification, water conservation, and soil management, can help farmers adapt to changing environmental conditions and improve their livelihoods.

These steps, along with other interventions, can help address the critical issue of farmer suicides in Maharashtra and improve the overall well-being of farmers in the region.

## Limitations

This study is subject to the following limitations:

- *Reliance on secondary data* The study utilizes government-released data (SCRB, PMFBY reports), which may have inherent issues such as underreporting or regional discrepancies in data collection processes.
- *Sensitivity to parameter selection* The predetermined selection of spatial (20%) and temporal (1–3 years) window parameters for spatiotemporal cluster detection presents a potential limitation of this study.

## Conclusion

This study primarily focuses on identifying spatiotemporal suicide clusters among farmers across districts in Maharashtra and examining the impact of PMFBY constraints on both clustered and non-clustered regions. PMFBY was introduced by the government to alleviate stress caused by crop failures and natural calamities, which are major contributors to farmer suicides. A spatiotemporal analysis conducted for the period from 2017 to 2021, using temporal windows of 1 to 3 years, reveals a notable variation in the spatial clustering patterns of farmer suicides between two phases. In Phase 1, the districts of Bhandara, Chandrapur, Gadchiroli, Gondiya, Nagpur, and Wardha in the Nagpur division, along with Amravati, Akola, and Yavatmal in the Amravati division, were identified as the primary and secondary hotspots for farmer suicides. In Phase 2, the primary and secondary clusters emerged at the districts of Aurangabad, Beed, Hingoli, Jalna, Latur, Nanded, Osmanabad, and Parbhani in the Aurangabad division, as well as Ahmednagar, Dhule, Jalgaon, and Nashik in the Nashik division. Logistic regression analysis highlights the impact of PMFBY constraints in shaping clustered and non-clustered regions. Key factors influencing cluster formation include the non-loanee status of farmers, the area insured, the insured sum, and the proportion of premium payments made by farmers, the state, and the Indian government during the scheme’s implementation phase compared to the operational phase. This study provides empirical evidence that the implementation of the Pradhan Mantri Fasal Bima Yojana (PMFBY) has had a positive impact on reducing farmer suicides in Maharashtra, particularly in regions previously identified as high-risk. By employing spatiotemporal analysis and logistic regression, we demonstrate that areas receiving greater benefits from the scheme, such as higher insurance coverage, premium contributions, and insured crop values, have experienced a shift from clustered to non-clustered zones over time. These results underline the importance of expanding and tailoring the PMFBY scheme based on regional vulnerability. To maximize its effectiveness, policymakers should focus on enhancing accessibility in districts with persistent distress, ensuring equitable enrollment across socio-economic groups, and addressing complementary issues like mental health support, financial awareness, and institutional credit access. In doing so, PMFBY can evolve into a more holistic risk-mitigation tool, guiding evidence-based interventions that not only secure agricultural livelihoods but also contribute meaningfully to suicide prevention efforts across rural India.

In a broader context, these findings align with global observations where agricultural communities are increasingly vulnerable to economic, climatic, and institutional stressors, resulting in elevated suicide rates. Countries such as Australia and the United States have reported similar outcomes, where financial insecurity, debt, and crop failure are leading contributors to farmer suicides. This underlines the necessity of resilient policy interventions tailored to region-specific risks. Given the dynamic nature of agrarian distress, we propose the formulation of a long-term spatiotemporal monitoring framework capable of integrating multiple variables, climatic, demographic, economic, and policy-based, to enable proactive decision-making. Such a framework would not only assist in identifying emerging high-risk regions in real time but also enhance the efficiency of targeted interventions. Moreover, the analytical approach adopted in this study, combining spatial scan statistics and logistic regression, can be extended to assess the impact of other agricultural policies such as the Minimum Support Price (MSP), debt relief schemes, and climate-resilient farming programs. This ensures a scalable and adaptable evaluation mechanism applicable across diverse geographies and policy landscapes. By identifying critical hotspots and evaluating policy effectiveness through robust statistical models, this research provides valuable insights that can inform data-driven strategies for suicide prevention, agricultural resilience, and improved welfare of farming communities in India and beyond.

## Data Availability

The raw dataset used for this study is freely available and can be found through the links “https://mahacid.gov.in/publications/crime-in-maharashtra” and “https://pmfby.gov.in/adminStatistics/dashboard”.

## Abbreviations

DAC & FW: Department of Agriculture, Cooperation and Farmers Welfare
F1: F-measure
GLM: Generalized linear models
LLR: Log-likelihood Ratio
LR: Logistic regression
MoA & FW: Ministry of Agriculture and Farmers Welfare
NCRB: National Crime Record Bureau
PMFBY: Pradhan Mantri Fasal Bima Yojana
PS: Precision score
RR: Relative risk
RS: Recall score
SCRB: State Crime Record Bureau
